# On the Influence of Emotion on Conflict Processing

**DOI:** 10.3389/fnint.2012.00042

**Published:** 2012-07-09

**Authors:** Philipp Kanske

**Affiliations:** ^1^Department of Social Neuroscience, Max Planck Institute for Human Cognitive and Brain SciencesLeipzig, Germany

## Introduction

The ability to show coherent goal-directed behavior, even in the presence of distraction, requires the detection and resolution of conflict, for example between opposing action tendencies. This defining feature of cognitive control has been extensively studied with experimental tasks like the Stroop, Simon, or Flanker paradigm (Stroop, 1935; Simon and Rudell, 1967; Eriksen and Eriksen, 1974), which also yielded the description of an underlying neural network for conflict detection and resolution (Fan et al., 2003; Ridderinkhof et al., 2004). Understanding the influence of emotion on this system is particularly important because they are intrinsically tied to one another in real life (Pessoa, 2008). Recent evidence seems to be contradictory with reports of facilitation and impairment effects of emotion on the processing of conflict. On a conceptual basis both accounts can be supported. It may be argued that keeping up goal-directed behavior is especially difficult in emotional situations because of their greater distracting potential. On the other hand, emotional stimuli are also signals of relevance for a situation that might require particularly efficient cognitive control (Scherer, 1994). In line with this latter view, Norman and Shallice (1986) suggested that emotion, along with other instances like novelty or error commitment, might trigger cognitive control processes. The present opinion article aims at elucidating these diverging views by specifying two critical factors – task relevance and individual differences – that result in an enhancing or hindering influence of emotion on the processing of conflict.

## Task Relevance

To study conflict processing, participants are typically given a certain goal related to a target stimulus and need to ignore other irrelevant information. The irrelevant information could be related to peripheral distractor stimuli (e.g., in the Flanker task) or to stimulus-response-associations (e.g., in the Simon task). If this information is incongruent with the given goal the resulting conflict needs to be detected and solved in order to show coherent behavior. A series of recent studies demonstrated that the speed, with which conflict is resolved in such tasks is enhanced, if the presented conflict stimuli are emotional, i.e., emotion speeds up cognitive conflict processing (Kanske and Kotz, 2010, 2011b). This effect is independent of emotional valence and observable for positive and negative stimuli alike (Kanske and Kotz, 2011a). It is also present in the visual and auditory modality (Kanske and Kotz, 2011d) and in Flanker, as well as Simon-type conflict tasks (Kanske and Kotz, 2011b,c). The latter is especially interesting as the mechanisms underlying Flanker and Simon tasks are very different. Conflict in the Simon task is not elicited through peripheral distractor stimuli, but through incompatible stimulus presentation and response side mapping. If, for example participants perform a voice gender discrimination with female voices requiring a right hand response, but the stimulus is presented on the left side, the elicited action tendencies will conflict and responses are prolonged. Even though this task does not require participants to focus on emotions, responses in the incompatible condition are accelerated if the spoken word is emotional (Kanske and Kotz, 2011b). The presence of the emotional modulation of conflict processing across these different conflict tasks and different sensory modalities strongly suggests that it is a general mechanism acting on the supra-modal processing steps of conflict detection and resolution rather than on visual or auditory spatial attention. Critically, however, in each of these studies the emotionally valent stimuli were task-relevant, i.e., the stimuli that participants needed to process and react to in order to solve the task. This contrasts other studies that modulated emotion through the presentation of additional stimuli that were unrelated to the task. For example, when emotional stimuli are presented before the conflict stimuli, conflict resolution is not facilitated, but impaired (Hart et al., 2010). Furthermore, conflict adaptation, i.e., the enhanced processing of conflict after presentation of an incongruent trial, is also compromised if emotional stimuli are presented between conflict trials (Padmala et al., 2011). This pattern of hindering and enhancing effects of emotion on conflict processing suggests that the exact role that emotion plays in a task is crucial for determining its impact. Emotion will only enhance cognitive control if the behaviorally relevant stimuli that participants react to during a task are emotional. Transiently induced emotion by task-unrelated stimuli, in contrast, yields hindering effects (for potentially conflicting results see Birk et al., 2011; Melcher et al., 2012, but see also Cohen and Henik, 2012, in this Research Topic for a discussion). Corroborating evidence for this conclusion also comes from two recent studies on the effects of motivation on conflict processing. Similarly to emotional stimuli that signal importance of a situation, if relevance is increased through reward for correct task performance, cognitive control is triggered resulting in accelerated conflict processing (Padmala and Pessoa, 2011). If, however, the motivational salience of the distractor stimuli is increased, conflict resolution is impaired (Krebs et al., 2010). It may thus be relevance in general that speeds up conflict processing, rather than emotion specifically.

## Neural Underpinnings

A crucial node in the neural network underlying cognitive control is the anterior cingulate cortex (ACC), in particular its dorsal portion (Ridderinkhof et al., 2004). The ventral ACC has been shown to be more sensitive to emotion (Bush et al., 2000) and also to conflict between emotional stimuli of different valence (Etkin et al., 2006). This ventral portion is also heavily connected to the amygdala, which is critically involved in emotion detection and generation (Phelps and LeDoux, 2005). In the context of emotional influences on conflict processing, interactions between these three regions seem important. Conflicting stimuli that are emotionally negative activate the ventral ACC in addition to the dorsal portion, which is active for incongruent stimuli irrespective of their emotional status (Kanske and Kotz, 2011b). Furthermore, functional connectivity between the ventral and dorsal ACC, as well as between ventral ACC and the amygdala is increased in incongruent emotional compared to neutral trials (Kanske and Kotz, 2011c). This suggests that the ACC is integrating the need for increased cognitive control in conflicting situations with task-relevant emotional stimuli. The emotional saliency information is signaled by the amygdala and the ACC prioritizes these situations resulting in enhanced conflict processing. The relative speed of this process is apparent in an emotional modulation of the first conflict-related component of the event-related potential of the EEG (Kanske and Kotz, 2011a). Already 200 ms after stimulus onset, emotional incongruent stimuli elicit an enhanced negativity over central electrodes, which has been localized to the dorsal ACC in non-emotional conflict processing (N200; van Veen and Carter, 2002). The effects of task-irrelevant emotional stimuli on reducing conflict processing efficiency are also mediated by the ACC. Activation reduction in this region during emotional distraction correlates with increased response times in the conflict task (Hart et al., 2010). The data on the neural underpinnings of reward-related influences on conflict processing show some similarities with the nucleus accumbens involved in behavioral facilitation of conflict resolution by reward associated stimuli, and a medial frontal region just dorsal to the ACC integrating information on the reward value of distractor stimuli (Krebs et al., 2011).

## Individual Differences

As individuals differ in sensitivity to emotional stimuli and cognitive control capabilities, there are also variations in the modulation of conflict processing through emotion. A recent correlational study examined this across six experiments on the influence of task-relevant emotional stimuli on conflict processing (Kanske and Kotz, 2012). Individuals high in subclinical depression and anxiety, both emotional states with deficient emotion processing (Kalia, 2005; Li et al., 2008), show a reduced increase in cognitive control for negative emotional stimuli. In contrast, the temperament trait effortful control, which correlates positively with conflict processing and describes the ability for self-regulation (Gerardi-Caulton, 2000) is associated with an enhanced emotion induced facilitation of conflict processing. These relations also translate to the neural level. High depression and anxiety are characterized by a smaller conflict-related increase in ventral ACC activity and N200 amplitude in task-relevant emotional stimuli, while effortful control has the opposite effects. Anxiety might also be related to the impairing effect of task-irrelevant emotional stimuli on conflict processing, but the evidence regarding the direction of this effect is rather mixed (Dennis and Chen, 2007; Dennis et al., 2008). The presence of these individual differences and their systematic relation to emotional states and temperament across different experimental designs (Kanske and Kotz, 2012) may therefore represent an important aspect when explaining the enhancing and hindering effects of emotion on cognitive control.

## Integration

**Figure 1:**
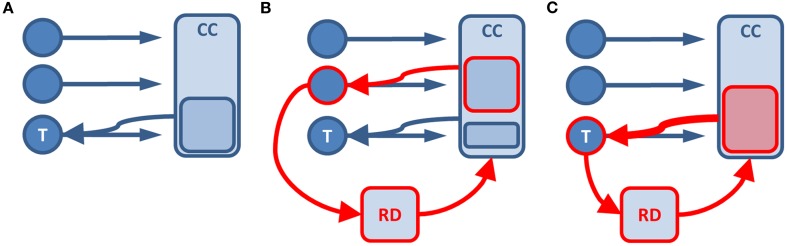
**Illustration of the functional integration of emotion and cognitive control in conflict processing**. The situation for neutral **(A)** and emotional task-irrelevant **(B)** or task-relevant stimuli **(C)** is displayed. Circles represent stimuli. Rectangles stand for processing units. Red frame color indicates an emotional stimulus or emotion induced processing. T, task-relevant stimulus; CC, cognitive control; RD, relevance detector.

The discussed results suggest a tight integration of emotion and cognitive control in situations that require the resolution of conflict. Figure 1 illustrates this for conflict processing in neutral stimuli (Figure 1A), and for an emotional task-irrelevant (Figure 1B) or task-relevant (Figure 1C) stimulus. In the well-described situation of conflict in neutral stimuli, cognitive control resources will be recruited and bias information processing in line with current task-demands through amplification of the cortical representation of the task-relevant stimulus (Egner and Hirsch, 2005). If a task-unrelated stimulus is emotional, relevance detectors like the amygdala or nucleus accumbens will divert resources toward the processing of that stimulus, thereby impairing the processing of task-related conflict (Pessoa, 2009). The ACC closely follows this pattern by showing emotion-related activation increase that predicts impaired task performance (Lim et al., 2008). ACC activity related to conflict, however, is decreased under emotional distraction, which also correlates with impaired behavior (Hart et al., 2010). In contrast, when the task-relevant stimulus is emotional, the current task-goal and emotional information signaled through the relevance detector coincide. Therefore, increased cognitive control resources will be recruited yielding an enhanced bias in information processing toward task-relevance. Here, the ACC also follows this pattern with the additional activation of the ventral portion for conflict in emotional stimuli, where the resolution of conflict is accelerated compared to neutral stimuli (Kanske and Kotz, 2011b,c). These interactions are variable, for example with individual differences in emotion-related amygdala reactivity and limbic-prefrontal connectivity in depression and anxiety (Johnstone et al., 2007; Kanske and Kotz, 2012). Here, the altered relevance detection seems to result in the dysfunctional integration of emotion and current task-goals apparent in reduced ventral ACC activation. This consequently leads to impaired performance.

## Challenges and Questions

The evident pattern might show some valence specificity. Even though negative and positive emotional stimuli yield comparable behavior and EEG results (Kanske and Kotz, 2010, 2011a), there is less data yet on the neural mechanisms underlying the influence of positive emotion on conflict processing. As depression and anxiety selectively affect the influence of negative, but not positive emotional stimuli on conflict processing (Kanske and Kotz, 2012), the mechanisms that mediate the influence on conflict processing might be different (see also Prehn et al., 2011). For positive emotion they might resemble more the pattern observed for reward-related stimuli with nucleus accumbens involvement (Krebs et al., 2011). The mechanisms might also differ for highly salient emotional stimuli, as suggested by Pessoa (2009). This notion is supported by the observed effects for anxiety and depression, which are characterized by increased sensitivity to negative emotional stimuli and increased related amygdala responding (Kanske and Kotz, 2012). The similarity of the results for motivation and emotion suggests that it may be relevance in general that yields enhanced conflict processing. This question should be followed up by direct comparisons of differently salient stimuli. Lastly, the relation of emotion and cognitive control is not uni-directional, rather, different levels of control may also affect emotion. There is some indication that individuals with high emotion regulation capacity attenuate emotional processing when cognitive control is recruited for conflict processing (Cohen et al., in press). The challenge for future investigations will be to concurrently study these reciprocal relations and their underlying neural mechanisms.

## Conclusion

Conflict processing is directly influenced by emotion, with individual differences in temperament and emotional state, as well as the task-relevance of the emotionally valent stimuli critically determining if this influence is an enhancing or a hindering one. The resulting pattern is highly evolutionary adaptive as the time that conflict yields an organism incapable of responding to potentially dangerous negative or reward-signaling positive stimuli is reduced. In contrast, if stimuli are present that are not relevant to current task-goals, but may still be relevant to higher order goals because of their danger- or reward-value, their processing is prioritized over task-performance. In consequence, the flexibility of behavior is greatly enhanced with slower and faster processing routes depending on current task-related and overarching goals.
